# Can digital finance reduce industrial pollution? New evidence from 260 cities in China

**DOI:** 10.1371/journal.pone.0266564

**Published:** 2022-04-14

**Authors:** Hongmei Wen, Jingliang Yue, Jian Li, Xuedan Xiu, Shen Zhong

**Affiliations:** 1 School of Finance, Harbin University of Commerce, Harbin, Heilongjiang, PR China; 2 Harbin Finance University, Harbin, Heilongjiang, PR China; Universiti Malaysia Sabah, MALAYSIA

## Abstract

Industrial pollution reduction is a crucial issue in the pursuit of sustainable economic and environmental development. As a product of the deep integration of traditional finance and Internet information technology, digital finance has become an effective tool for regulating the use of funds and strengthening the effectiveness of policies in the context of the digital era, which has obvious effects on industrial pollution emissions. Using panel data of 260 prefecture-level cities in China from 2011–2019 and the digital inclusive finance index jointly compiled by Peking University and Ant Financial Services Group, this paper empirically analyzes the impact of digital finance on industrial pollution emissions through fixed effects model, mediating effects model and threshold effects model. The empirical results show that digital finance can effectively reduce industrial pollution and part of the impact is achieved through industrial structure. In the process of reducing industrial pollution by digital finance, there exists double threshold effects. When the development of digital finance breaks the threshold value, the industrial pollution emission reduction effect appears to accelerate. Finally, this paper puts forward targeted suggestions to promote industrial pollution reduction and environmental economic development.

## 1. Introduction

In the 21st century, along with economic prosperity and technological progress, environmental problems have brought threats and hazards to mankind. Global ecological problems have attracted the attention of governments and people around the world. China, which has been developing rapidly for more than 40 years after reform and opening up, is considered internationally to be in a period of high pollution and high emissions. Seeking sustainable economic and environmental development is now a crucial issue.

Economic development cannot be achieved without industrial activities, however, pollution formed in industrial activities has a great impact on the environment and human beings. Taking sulfur dioxide and nitrogen oxide in industrial pollution as an example, according to the 2018 China Environmental Statistics Yearbook, industrial sulfur dioxide accounts for 86.55% of total sulfur dioxide emissions, besides, industrial nitrogen oxide accounts for 45.69% of total nitrogen oxide emissions (China Environmental Statistics Yearbook, 2018). Meanwhile, sulfur dioxide and nitrogen oxide in the atmosphere can cause acid deposition, which can lead to transnational pollution with atmospheric movement and cause surface ecological environment acidification. The formation of sulfuric acid fog can directly invade human lungs causing lethal diseases such as pulmonary edema [1].

In the economic field, factors that influence industrial pollution emissions include economic development, labor force, foreign direct investment, scientific and technological innovation, environmental regulation and financial development [2–6]. Among them, finance plays a key role in the process of industrial pollution reduction as an effective tool to regulate the financial situation, promote income growth and strengthen policy effectiveness. With the continuous development of digital technology and the deep integration of traditional finance with the information technology, China’s financial industry has fully entered the digital finance era. Digital finance provides more channels, more levels and more forms of financial services, establishing a more balanced investment and financing environment for industrial enterprises, which in turn provides essential financial support for industrial pollution abatement [7].

In China, digital finance as a whole shows a trend of steady development, but there are still certain differences among different regions, which, as a kind of regional resource endowment, may cause industrial transfer and affect industrial structure optimization; in addition, due to the differences in the stage characteristics of the development process of digital finance, the impact on industrial pollution emissions may produce structural abrupt changes or jump changes. That means a non-linear relationship between digital finance and industrial pollution emission is possible which makes the relationship more complex. Therefore, it is important to fully understand the role of digital finance and the influence mechanism so as to take advantage of digital finance rationally and promote sustainable economic development.

This paper selects panel data of 260 prefecture-level cities in China from 2011–2019 to study the impact of digital finance development on industrial pollution emissions and refines the impact mechanism through the mediating effects generated by industrial structure optimization. Finally, using threshold regression, this paper explores whether there is a sudden structural change or jump change in the industrial pollution emission reduction effect of digital finance. The remainder of this paper is organized as shown below: Section 2 reviews the relevant literature; Section 3 introduces the selection of models, variables and data sources; Section 4 reports the empirical results; Section 5 provides a general discussion; Section 6 contains conclusions and recommendations.

## 2. Literature review

Research on the relationship between the environment and economic activities first appeared in 1971, when Ehrlich and Holdren discussed the impact of human based on social and economic activities on ecological environment. On this basis, they proposed the IPAT model, which considers the ecological environment as a function of population size, technological innovation and the level of economic development, where the regional population is considered as the most important factor affecting the ecological environment [8]. However, the IPAT model assumes that the environment changes in the same proportion as its influencing factors. Dietz, Rosa, and York (2003) addressed this shortcoming by proposing a stochastic form of the IPAT equation, the STIRPAT model, which not only tests the elasticity coefficients of the influence of each factor on environmental pollution, but also extends other factors that have an impact on environmental pollution [9]. In 1992, Grossman and Krueger empirically analyzed the relationship between economic development and environmental pollution in the United States and concluded that the relationship is in an "inverted U-shape", i.e., environmental stress increases with economic growth and then decreases slowly with economic development [10]. Panayotou (1993) referred to the "inverted U-shaped" relationship between environmental pressure and economic development as the environmental Kuznets curve, also known as the EKC curve, based on the Kuznets curve theory [11].

With the continuous research on environmental issues, the influencing factors affecting industrial pollution emissions have become hot research topics in recent years. Scholars have discussed about the influence of economy, labor force and technology on industrial pollution based on IPAT theory and environmental Kuznets curve, while more influencing factors have been included in the discussion, such as foreign real investment, financial development, industrial structure and so on [2–6, 12]. Among them, finance plays a key role in the reduction of industrial pollution as an effective tool to regulate the financial situation, promote income growth and enhance policy effectiveness.

As a crucial part of economic activities, the impact of finance on the environment has also attracted the attention of scholars. Some scholars in the existing literature have argued how financial development exacerbates environmental pollution and some other factors that may affect the phenomenon, while more scholars, after empirical studies, have drawn an opposite conclusion [13, 14]. Samples of such as Saudi Arabia, Africa, China, France and 88 developing countries have been selected to study the relationship between financial development and the environment pollution [15–19]. It was found that financial development can reduce pollutant emissions and help energy conservation by establishing city commercial banks and attracting low pollution FDI inflows [20–23].

Regarding the relationship between finance and industrial pollution emissions, many scholars believe that financial development is beneficial to industrial pollution reduction. By studying the impact of financial development on industrial pollution emissions in a number of large national industries, Haas and Popov (2018) found that financial development can direct more investment to cleaner industries and promote more green patents with the development of stock markets [24]. Examining provincial data from China, Karl and Chen (2010) found that in addition to the measures above, financial development can also improve environmental performance through income and regulation effects [25].

There is less information on the relationship between digital finance and industrial pollution while scholars have focused more on the impact of digital finance on economic growth, innovation and entrepreneurship. Economically, digital finance can promote economic growth by reducing the income gap and the breadth of coverage can reduce the urban-rural income gap more significantly compared to other dimensions of digital inclusive finance [26, 27]. Meanwhile, digital finance can promote economy by increasing residential consumption especially the recuring household expenditure [28, 29]. Household leverage, which may be related to consumption, was also studied by scholars. For example, Wang et al. (2021) analyzed the impact of digital finance on household indebtedness by way of influencing household consumption and liquidity [30]. Results show that digital finance can alleviate the over-indebtedness of Chinese households. While in the study of Huang (2021), digital financial development has significantly increased the leverage of Chinese households [31]. In terms of innovation and entrepreneurship, Digital finance is a clear contributor in both respects. Specifically, although digital finance has no effect on entrepreneur-oriented entrepreneurship, but could spur the liberation of regional entrepreneurship, especially in the regions which are characterized by a lower urbanization rate and lower physical capital [32, 33]. In the central and west region, labor-intensive industries, high-tech sector and private-owned companies, digital finance increased the intensity of innovation and enhanced the level of digitalization, so that manufacturing servitization was promoted [34].

In the existing literature, the threshold effects model and the mediating effects model have been widely used. For example, in the study of measuring the green total factor productivity of China’s provinces under resource and environmental restrictions, financial development was chosen as a threshold dependent variable which had a non-linear, double-threshold effect on green total factor productivity and diminishing marginal efficiency [23]. Li et al. (2020) examined impacts of the digital inclusive finance on household consumption and explored its mechanisms, the results indicated that online shopping, digital payment, obtainment of online credit, purchase of financing products on the internet and business insurance were the main mediating variables through which digital finance affected household consumption [29].

In terms of data, most scholars selected provincial data as their research samples. For example, Zhao et al. (2019) selected data from 30 Chinese provinces to study how economic growth, energy consumption and financial development impact on environmental pollution; Zhou et al. (2019) used provincial panel data to investigate the green total factor productivity of China’s provinces under environmental restrictions; Xu (2019) analyzed the impacts of China’s financial development on environmental pollution, using the provincial panel data of China [6, 17, 20].

In general, most scholars believe that finance can play a suppressive role in environmental pollution and further discuss the mechanism of financial impact on the environment. Mainly mediating effects and threshold effects are included. However, the existing literature has ignored the background of the era of deep cooperation between traditional finance and fintech enterprises and has not paid enough attention to the far-reaching impact of digital finance on financial resource allocation when studying the relationship between financial development and environmental pollution; in addition, little literature has paid attention to the mediating effects of industrial structure in the process of financial reduction of environmental pollution. Finance, as an important market-based mechanism, plays a key role in optimizing the structure of the economy and promoting the healthy development of the industrial structure. The optimization of the industrial structure is naturally and closely related to the reduction of industrial pollution and the merits of the industrial structure determine the level of energy consumption and environmental protection of the economy.

The possible innovations of this paper are as follows: (1) Digital financial development is used as the core explanatory variable to explore its impact on industrial pollution reduction, considering the background of the times when technology deeply empowers the financial industry. (2) Using industrial structure as a mediating variable, the mechanism of digital financial development’s impact on industrial pollution is explained. (3) The instrumental variable method is chosen to solve the endogeneity problem in the regression and make the estimation more robust.

## 3. Models and data

In this section, 3.1 explains the selection and establishment of the basic model, mediating effects model and threshold effects model; 3.2 introduces the meanings of variables, data and data sources. 3.3 illustrates how industrial pollution and digital finance changed during the sample period with maps.

### 3.1 Model setting

In order to study the impact of digital finance development on industrial pollution reduction, explore whether industrial structure optimization has mediating effects in this process and whether there is a sudden structural change or jump change in the impact of digital finance on industrial pollution, the following models are set up in this paper, where 3.1.1 is the basic model, 3.1.2 is the mediating effects model and 3.1.3 is the threshold effects model.

#### 3.1.1 Basic model

To test whether digital finance reduces industrial pollution, this paper selects panel data of 260 cities in China from 2011–2019 and first uses pooled regressions with the following expressions.

$$pol_{it}=a+\beta_{1}df_{it}+\gamma X_{it}+\delta z_{i}+\varepsilon_{it}$$ (1)

where *i* represents the city and *t* represents time; *pol* is the degree of industrial pollution represented by total industrial pollution emissions per square kilometer; *df* is the digital financial inclusion development index; X is a series of control variables, including *lnpgdp*, *lnpopu*, *inno*, *edu* and *lnfc*, which respectively denote economic growth, population density, technological innovation, educational attainment and offshore investment; *z*_*i*_ represents individual characteristics that do not change over time; *a* is a constant term and *ε*_*it*_ is the random disturbance term. The pooled regression assumes that all individuals have exactly the same regression equation and there are no individual effects, for which the assumption must be tested statistically. Therefore, the fixed-effects model and the random-effects model are estimated separately in this paper where the expressions of the fixed-effects model are as follows.


$$pol_{it}=\beta_{2}df_{it}+\gamma X_{it}+\delta z_{i}+u_{i}+\varepsilon_{it}$$ (2)

The meaning of the variables in the expression is the same as in Eq (1), but the perturbation terms are given by *u*_*i*_ and *ε*_*it*_ two components: *u*_*i*_ is an unobservable intercept term representing individual heterogeneity and is associated with some explanatory variable; *ε*_*it*_ is a nuisance term that varies with individuals and time. If *u*_*i*_ is not correlated with all explanatory variables, it is called a random effects model and the expression is as follows.


$$pol_{it}=\beta_{3}df_{it}+\gamma X_{it}+\delta z_{i}+u_{i}+\varepsilon_{it}$$ (3)

In this paper, the F-test, LM-test and Hausman test will be used to verify whether there is an individual effect and whether *u*_*i*_ is correlated with the explanatory variables in order to select the basic research model for this paper.

#### 3.1.2 Mediating effects model

Digital finance can significantly contribute to economic development and reduce the income gap as a way to guide the path of labor transfer from primary to secondary and eventually tertiary industries [26, 27, 35]. In addition, not only can digital finance increase national innovation and entrepreneurship, but also adjust the input and output efficiency and resource allocation of industries from both supply and demand sides [36]. Thus, digital finance adjusts the industrial structure and influences the upgrading through economic growth, entrepreneurship and innovation. Since the industrial structure determines the energy consumption level and environmental protection level of the economy, which are naturally and closely related to industrial pollution reduction, digital finance may have reduced industrial pollution emissions by optimizing the industrial structure. In order to test the mediating effects, this paper uses industrial structure as a mediating variable to study the intrinsic mechanism of digital finance development acting on industrial pollution emission. Drawing on Wang Peng et al. (2019), the degree of industrial structure optimization is measured by the ratio of tertiary industry to secondary industry (TS), and the following test model is set [37].


$$TS_{it}=\beta_{1}df_{it}+\gamma X_{it}+\delta z_{i}+\varepsilon_{it}$$ (4)

$$pol_{it}=\beta_{2}df_{it}+\beta_{21}TS_{it}+\gamma X_{it}+\delta z_{i}+\varepsilon_{it}$$ (5)

Eqs (4) and (5) use TS as a mediating variable to test the impact of digital finance development on industrial pollution reduction through industrial restructuring.

#### 3.1.3 Threshold effects model

Digital finance initially emerged in China in the form of Internet-enabled traditional financial institutions, after which fintech companies took advantage of their massive user base to make digital finance users grow significantly. Finally, traditional finance immensely increased the depth of digital finance usage through in-depth cooperation with fintech companies [7]. Digital finance at different stages of development has different characteristics, which may lead to structural abrupt changes or jumps in the impact of digital finance on industrial pollution emissions, resulting in a nonlinear relationship between them. In order to make the estimated values of the research coefficients more stable, this paper draws on Hansen (1999) and uses digital financial development as a threshold variable to discuss whether there are sudden structural changes or jumps in digital financial development and whether its impact on industrial pollution takes another form when it reaches a certain value [38]. The single threshold regression expression for the panel data is as follows.


$$pol_{it}=a+\beta_{11}df_{it}I\left(df_{it}\leq \lambda \right)+\beta_{12}df_{it}I\left(df_{it}>\lambda \right)+\gamma X_{it}+\varepsilon_{it}$$ (6)

*df is* both the core explanatory variable and the threshold variable; *λ* is the threshold value to be estimated and *I(·)* is the indicative function that takes on a value of 1 when the conditions in parentheses are met and 0 otherwise.

Alternatively, if there is a situation where more than one threshold exists, the expression for a double threshold, as an example, is as follows

$$pol_{it}=a+\beta_{11}df_{it}I\left(df_{it}\leq \lambda_{1}\right)+\beta_{12}df_{it}I\left(\lambda_{1}<df_{it}\leq \lambda_{2}\right)+\beta_{13}df_{it}I\left(df_{it}>\lambda_{2}\right)+\gamma X_{it}+\varepsilon_{it}$$ (7)

### 3.2 Data and data sources

1. Industrial pollution. In this paper, the total emissions (pol) of industrial wastewater (wwater, unit:t), industrial sulfur dioxide (so2 unit:t) and industrial dust (dust unit:t) per unit area (per square kilometer) of the city are selected to describe the degree of industrial pollution and are used as the explained variable.
2. Digital financial development. This paper selects the China Digital Financial Inclusion Index jointly compiled by Digital Finance Research Center of Peking University and Ant Financial Services Group to measure the degree of digital financial development in China. The index is compiled on the basis of the big data obtained from the transaction accounts of Ant Financial Services and consists of the breadth of coverage, depth of use and degree of digital support services: the breadth of coverage is described by the number of electronic accounts; the depth of use is measured by the actual use of Internet financial services; and the degree of digital support services is reflected by convenience and low cost. The specific compilation system is shown in Table 1 in the attached table. This paper uses the index divided by 100(df) as the core explanatory variable. df1, df2 and df3 are used to describe the breadth of coverage, depth of use and degree of digital support services of digital financial services, respectively.

**Table 1:** Variable selection and definition.

Variable Name	Variable Meaning	Metrics
pol	Industrial Pollution	The sum of industrial wastewater, industrial sulfur dioxide and industrial dust emissions per square kilometer
df	Digital Financial Development	Digital Inclusive Finance Index/100
lnpgdp	Economic Growth	Logarithm of gdp per capita
lnpopu	Population Density	Logarithmic value of population per square kilometer
Innovation	Technology Innovation	Innovation and Entrepreneurship Index Total Index Score
Lnfc	Offshore Investment	Logarithm of the actual amount of foreign capital used in the current year
Edu	Education Level	Number of general higher education teachers per 10,000 people
3. Control variables. In the IPAT model proposed by Ehrlich and Holdren, the ecological environment is regarded as a function of population size, technological innovation and economic development level, in which the impact of economy on environment is also reflected in the environmental Kuznets curve. Therefore, the logarithm of per capita gdp (lnpgdp unit: yuan), the logarithm of population density (lnpopu unit: person/square kilometer) and the total index score of the innovation and entrepreneurship (innovation) compiled by the Enterprise Big Data Research Center of Peking University are selected as proxy variables for economic growth, population density and technological innovation, respectively. In addition, based on the "pollution paradise hypothesis" and the "pollution halo hypothesis", overseas investment may affect the environmental quality of the host country [4]. The logarithm of the actual amount of foreign investment (lnfc) used in the year (unit: ten thousand dollars) is chosen to represent the extent of overseas investment. Meanwhile, the level of education affects the environmental awareness of residents which may impact on industrial pollution emissions [39]. Therefore, this paper includes the level of local education as an influencing factor of industrial pollution and measures the level of education in terms of the number of general higher education teachers per 10,000 people (edu unit: people per 10,000 people).

The data in this paper, except for the "Peking University Digital Inclusive Finance Index" and "China Regional Innovation and Entrepreneurship Index", are obtained from the China City Statistical Yearbook, with the sample time span of 2011–2019. Table 1 shows the selection and definition of variables and Table 2 shows the descriptive statistics of variables.

**Table 2:** Descriptive statistics of variables.

Variable	Obs	Mean	Std. Dev.	Min	Max
pol	2340	8.194	13.269	0	259.857
wwater	2340	.745	1.439	0	19.976
so2	2340	4.234	6.043	0	66.49
dust	2340	3.216	9.626	0	254.935
df	2340	1.661	.653	.213	3.216
df1	2340	1.564	.631	.045	3.109
df2	2340	1.645	.677	.125	3.32
df3	2340	2.01	.818	.027	4.379
lnpgdp	2340	10.734	.572	9.091	15.675
lnpopu	2340	5.794	.871	1.513	7.923
lnfc	2340	10.006	1.887	0	14.544
innovation	2340	52.186	28.32	.342	100
edu	2340	10.581	14.03	0	83.467

Table 2 shows the descriptive statistics of variables. It can be seen that, there are less fluctuations in the data of digital finance compared to industrial pollution, indicating a more balanced and smooth trend development of digital finance.

### 3.3 Data characteristics

After analyzing the panel data, it can be seen that industrial pollution emissions have gradually decreased and digital financial development levels have gradually increased during 2011–2019. This trend is more evident in the central and eastern region compared to the western region.

## 4. Empirical results

Through panel data regression, mediating effects regression and threshold effects regression, this paper analyzes the impact of digital finance development on industrial pollution with an empirical approach. 4.1 describes the industrial pollution emission reduction effect of digital finance development using panel data regression. 4.2 describes the impact from different dimensions. 4.3 analyzes the impact of digital finance on different industrial pollutant emissions. 4.4 uses industrial structure as a mediating variable so as to analyze how digital finance helps industrial pollution reduction through the optimization of industrial structure. 4.5 uses threshold effects regression to analyze whether there are sudden structural changes or jump changes in the process of digital finance emission reduction. 4.6 analyzes the robustness.

### 4.1 Impact of digital finance on industrial pollution emissions

Table 3 reports the regression results of the panel data. The three models are pooled regression, fixed-effects regression and random-effects regression, respectively. Referring to the test results of the F-test, LM-test and Hausman test reported in the table, the fixed-effects regression model should be selected as the basic research model of this paper.

**Table 3:** Basic regression results.

	(1)	(2)	(3)
	OLS	FE	RE
df	-7.408***	-5.969***	-6.584***
	(0.456)	(0.466)	(0.421)
lnpgdp	6.618***	2.370**	4.412***
	(0.723)	(1.085)	(0.881)
lnpopu	3.724***	10.549*	3.505***
	(0.337)	(5.754)	(0.592)
innovation	0.057***	0.054**	0.068***
	(0.014)	(0.023)	(0.018)
lnfc	-0.609***	-0.126	-0.289
	(0.181)	(0.245)	(0.213)
edu	-0.028	-0.108	-0.026
	(0.022)	(0.105)	(0.037)
_cons	-68.719***	-68.851**	-48.890***
	(7.126)	(34.034)	(9.065)
Obs.	2340	2340	2340
FM test		5.47***	
LM test			990.76***
Hausman test		17.04**	

Standard errors are in parenthesis.

*** p<0.01,

** p<0.05,

* p<0.1.

According to the regression results of model (2), we can see that the coefficient of digital finance (df) is -5.759 and significant at the 1% level, which empirically indicates that digital finance has a significant effect on industrial pollution reduction. The control variables of economic level per capita (lnpgdp), population density (lnpopu) and technological innovation (inno) are all significantly positive at least 10% level, reflecting the promotion effect on industrial pollution emission, which is consistent with the IPAT theory mentioned before. Economic development and population expansion will bring more pollution. Moreover, the application of science and technology leads to the over-exploitation of resources. Further, the environmental degradation occurs. The regression coefficients of overseas investment (lnfc) and education level (edu) are negative, but do not pass the significance test.

### 4.2 The impact of digital finance on industrial pollution emissions in different dimensions

Since the digital finance index can be measured from three dimensions: breadth coverage (df1), depth of use (df2) and degree of digital support services (df3), this section uses the indices of these three dimensions as core explanatory variables, respectively, and adopts a fixed-effects regression approach to analyze the industrial pollution reduction effect of digital finance in three dimensions.

**Fig 1:**
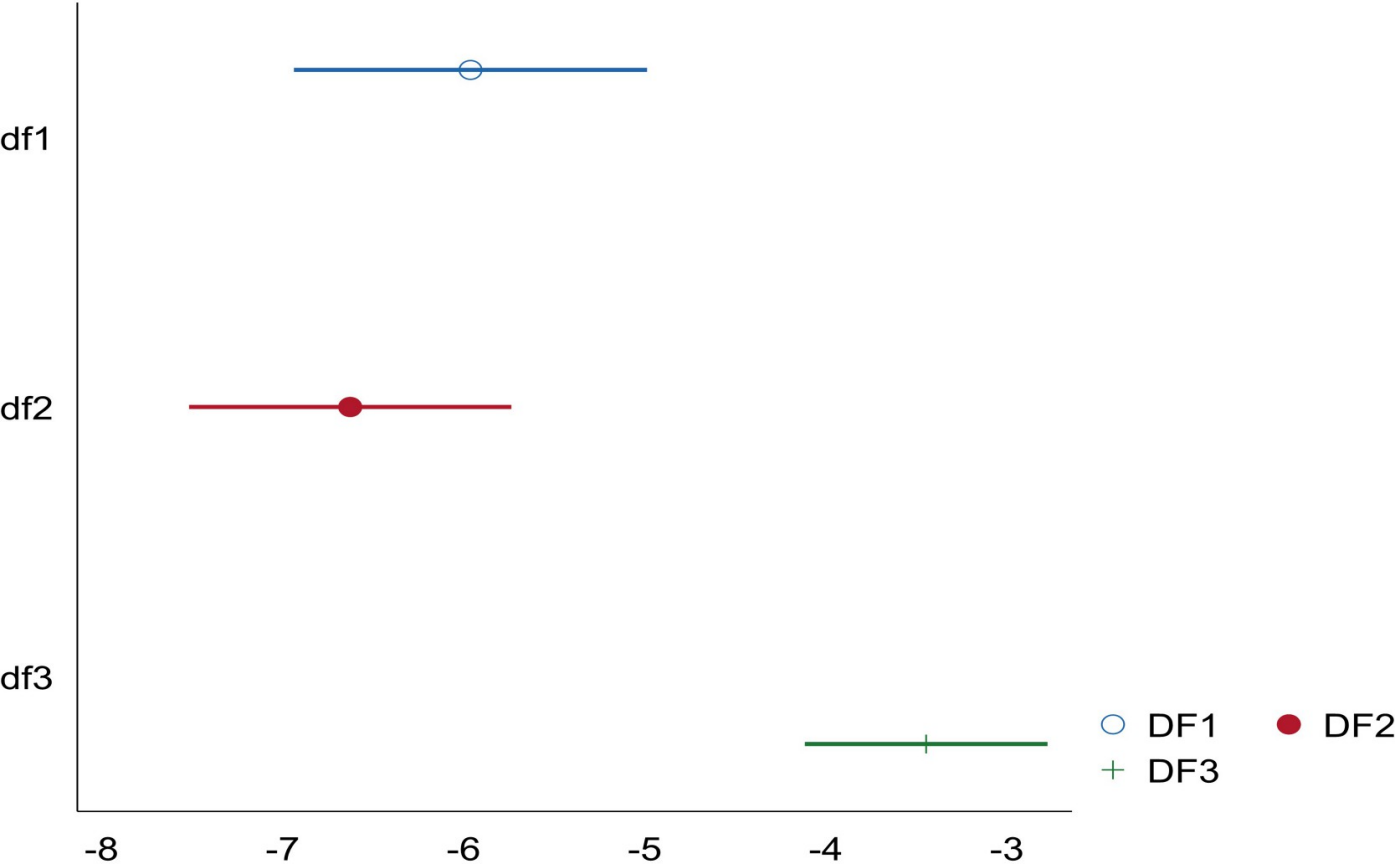
Marginal effects of df1, df2 and df3 on pol.

Columns (1) (2) (3) in Table 4 report the regression results with breadth of digital finance coverage (df1), depth of use (df2) and degree of digital support services (df3) as the core explanatory variables, respectively. It can be seen that the coefficients of df1, df2 and df3 are all significantly negative at the 1% level. As shown in Fig 1, compared to breadth of coverage (df1) and degree of digital support services (df3), the absolute value of the coefficient of depth of use (df2) is larger and the effect of industrial pollution reduction is stronger. It can be concluded that the depth of use of digital finance plays a more significant role in the process of industrial pollution reduction.

**Table 4:** Regression results of fixed effects for different dimensions of digital finance.

	(1)	(2)	(3)
	pol	pol	pol
df1	-5.964***		
	(0.497)		
df2		-6.628***	
		(0.454)	
df3			-3.448***
			(0.342)
lnpgdp	1.468	3.986***	-0.479
	(1.071)	(1.092)	(1.039)
lnpopu	10.743*	10.216*	8.061
	(5.785)	(5.683)	(5.826)
innovation	0.057**	0.047**	0.059***
	(0.023)	(0.022)	(0.023)
lnfc	-0.059	-0.288	0.093
	(0.246)	(0.244)	(0.247)
edu	-0.105	-0.119	-0.167
	(0.106)	(0.104)	(0.107)
_cons	-61.785*	-81.213**	-28.697
	(34.203)	(33.571)	(34.128)
Obs.	2340	2340	2340
R-squared	0.110	0.137	0.093

Standard errors are in parenthesis

*** p<0.01,

** p<0.05,

* p<0.1.

Analyzing the reason, it may be because the coverage breadth of digital finance reflects more the fairness of digital finance, so that less economically developed regions can reach financial services and reduce the uneven allocation of financial resources. But the essence of digital finance is still finance and its function needs to be realized through real economic activities. Depth of use, as a reflection of the results of digital finance application, describes the specific financial functions played by digital finance in economic activities. Only when digital finance really serves industrial enterprises can it reflect its inhibiting effect on industrial pollution emission. The degree of digital support, as a reflection of the low threshold and low-cost characteristics of digital finance, can increase the demand for financial services, but its industrial pollution emission reduction effect is relatively small compared to the breadth and depth of digital finance applications.

### 4.3 Impact of digital finance on the emission of different industrial pollutants

In the previous paper, industrial pollution is expressed as the sum of industrial wastewater (wwater), industrial sulfur dioxide (so2) and industrial dust (dust) emissions per unit area. In order to identify the variability of the impact of digital finance on different types of pollutants, this section analyzes the industrial pollution abatement effect of digital finance using fixed effects regression with each pollutant as the explained variable, respectively. Columns (1) (2) (3) in Table 5 report the regression results with industrial wastewater (wwater), industrial sulfur dioxide (so2) and industrial dust (dust) as explained variables, respectively. It can be seen that the coefficients of df are all significantly negative at the 1% level, indicating that digital finance has a significant inhibitory effect on the emissions of all three major industrial pollutants.

**Table 5:** Regression results of fixed effects for different pollutants.

	(1)	(2)	(3)
	wwater	so2	dust
df	-0.338***	-3.947***	-1.681***
	(0.023)	(0.154)	(0.421)
lnpgdp	0.137***	1.654***	0.579
	(0.053)	(0.358)	(0.979)
lnpopu	-0.162	6.681***	3.989
	(0.278)	(1.900)	(5.192)
innovation	0.001	0.020***	0.032
	(0.001)	(0.007)	(0.020)
lnfc	-0.016	0.015	-0.125
	(0.012)	(0.081)	(0.221)
edu	0.019***	0.009	-0.137
	(0.005)	(0.035)	(0.095)
_cons	0.660	-46.996***	-22.274
	(1.646)	(11.241)	(30.711)
Obs.	2340	2340	2340
R-squared	0.146	0.338	0.016

Standard errors are in parenthesis.

*** p<0.01,

** p<0.05,

* p<0.1.

### 4.4 Mediating effects

In order to test the mediating effects of industrial structure in the process of digital finance industrial emission reduction, the mediating effects model is selected for verification in this paper. The ratio of the output value of tertiary industry to the output value of secondary industry (TS) is used as the mediating variable and the method of Wen (2004) is borrowed to conduct the mediating effects test [40]. The regression results are shown in Table 6.

**Table 6:** Mediating effects regression results.

	(1)	(2)	(3)
	pol	TS	pol
df	-7.408***	0.435***	-6.740***
	(0.456)	(0.016)	(0.521)
TS			-1.535***
			(0.581)
lnpgdp	6.618***	-0.345***	6.089***
	(0.723)	(0.026)	(0.749)
lnpopu	3.724***	-0.132***	3.521***
	(0.337)	(0.012)	(0.346)
innovation	0.057***	0.000	0.058***
	(0.014)	(0.001)	(0.014)
lnfc	-0.609***	0.008	-0.598***
	(0.181)	(0.006)	(0.181)
edu	-0.028	0.016***	-0.004
	(0.022)	(0.001)	(0.024)
_cons	-68.719***	4.461***	-61.870***
	(7.126)	(0.253)	(7.575)
Obs.	2340	2340	2340
R-squared	0.183	0.354	0.186

Standard errors are in parenthesis

*** p<0.01,

** p<0.05,

* p<0.1.

According to the results reported in Table 6, the coefficients of digital finance (df) in columns (1) (2) and industrial structure (TS) in column (3) are significant at the 1% level, indicating that at least part of the impact of digital finance on industrial pollution reduction is achieved through industrial structure (TS). The significant coefficient of digital finance (df) in column (3) can confirm the mediating effect of industrial structure as a mediating variable. The sobel test is not required and the proportion of the mediating effects is 0.09.

### 4.5 Threshold effects

In order to test whether this effect has a sudden structural change or jump with the development of digital finance and to ensure the stability of the coefficients, the data are then analyzed using a panel threshold regression. In this section, using digital finance as the threshold variable, we used Stata 16.0 statistical software and borrowed Hansen’s (1999) "self-help method" to determine whether there are threshold effects by repeatedly sampling 300 times to obtain the p-value of the test statistic [38]. The test results are shown in Table 7.

**Table 7:** Threshold effects significance test.

Threshold	RSS	MSE	Fstat	Prob	Crit10	Crit5	Crit1
Single	1.94e+05	83.1316	72.79	0.0000	18.9760	21.6075	28.2881
Double	1.91e+05	82.1394	28.16	0.0133	17.6543	22.0361	30.2856
Triple	1.90e+05	81.4073	20.96	0.2133	29.9483	39.3406	71.0409

From the results in Table 7, it is clear that the F statistic is significant at least at the 5% level in the single threshold and double threshold models, however, the F value is not significant in the triple threshold model, indicating that there are double threshold effects in this model.

**Fig 2:**
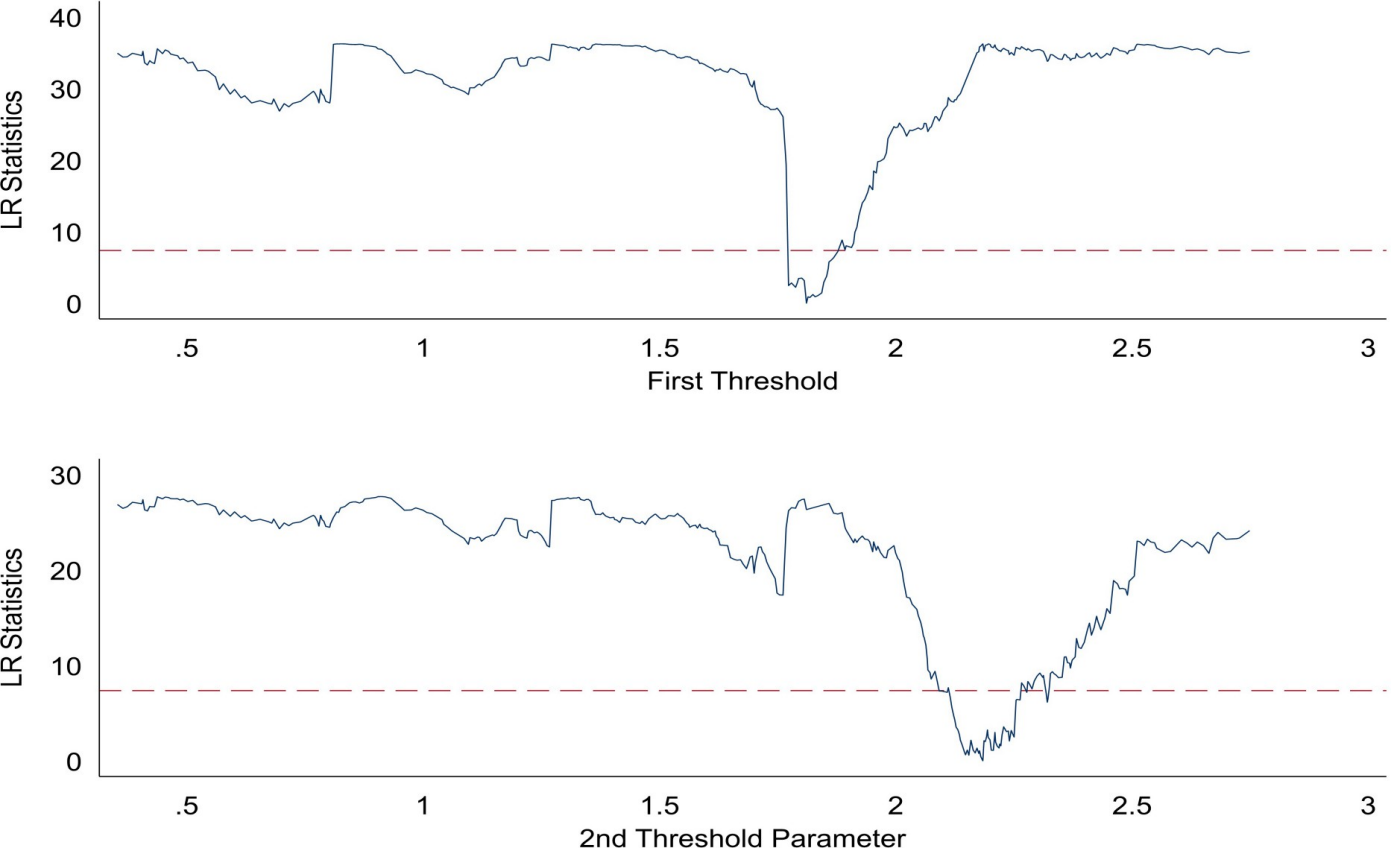
The construction of confidence intervals.

The results of the threshold estimates are given in Table 8, which are 1.8110 and 2.1843, respectively. Fig 2 shows the graph of the likelihood ratio function at 95% confidence interval for each of the two thresholds. The dashed line indicates the critical value of 7.35, which is significantly larger than the threshold value, indicating that the threshold value is truly valid.

**Table 8:** Threshold estimation results.

model	Threshold	95% CI	
Th-1	1.8433	1.8180	1.8476
Th-21	1.8110	1.7872	1.8142
Th-22	2.1843	2.1456	2.1868

Table 9 reports the results of the threshold regression and divides the sample into three subsamples by double thresholds for the staged regression. The regression coefficient of digital finance (df) is significantly negative at least at the 10% level in all three stages. When the value of digital finance (df) is less than or equal to 1.8110, its impact coefficient on industrial pollution is -1.230 and significant at the 10% level, indicating that when the development of digital finance is low, it can reduce industrial pollution to a certain extent; when the value of digital finance (df) is between 1.8110 and 2.1843, the impact coefficient is -3.377 and significant at the 1% level significant, indicating that when digital finance is further developed, it can significantly reduce industrial pollution; when digital finance (df) takes a value greater than 2.1843, the impact coefficient is -4.880, indicating that when digital finance is developed to a higher level, there is a significant and further increase in the level of suppression of industrial pollution emissions. In general, there are two bottlenecks in the process of reducing industrial pollution through digital finance. When the bottleneck is broken, its industrial pollution emission reduction effect appears to accelerate. The reason for this phenomenon may be related to the differences in the stage characteristics of the development process of digital finance.

**Table 9:** Double threshold effects regression results.

	(1)	(2)
VARIABLES	single	double
lnpgdp	2.939***	2.766***
	(1.071)	(2.60)
lnpopu	9.585*	11.749**
	(5.669)	(2.08)
innovation	0.059***	0.065***
	(0.022)	(2.96)
lnfc	-0.366	-0.382
	(0.244)	(-1.58)
edu	-0.087	-0.063
	(0.104)	(-0.103)
df(df≤1.8433)	-1.950***	
	(0.679)	
df (df> 1.8433)	-4.755***	
	(0.483)	
df(df≤1.8110)		-1.230*
		(0.701)
df(1.8110<df≤2.1843)		-3.377***
		(0.546)
df(df> 2.1843)		-4.880***
		(0.486)
Constant	-71.312**	-83.276**
	(33.524)	(33.442)
Observations	2,340	2,340
R-squared	0.145	0.155
Number of code	260	260

Standard errors are in parenthesis.

*** p<0.01,

** p<0.05,

* p<0.1.

At the early stage of digital finance development, the breadth of coverage, depth of use and degree of digital support are low, the application of digital finance by industrial enterprises is not common and the industrial emission reduction effect is not obviously reflected. When digital finance develops to the intermediate stage, with the number of digital finance users gradually rising, the application of digital finance by industrial enterprises becomes more common. The level of industrial pollution emission reduction is obviously improved. When digital finance develops to the advanced stage, the application of digital finance provided for industrial enterprises are more diversified and the scale of financing is expanded. The application of digital finance is deepened compared with the previous stage, thus further improving the level of suppression of industrial pollution emission by digital finance.

In this section, based on the results obtained from the threshold regression, the digital finance development is divided into three stages, namely, the primary stage of digital finance development, the intermediate stage of digital finance development and the advanced stage of digital finance development. The effects of the three dimensions at different stages are analyzed separately. The regression coefficients are shown in the following Table 10.

**Table 10:** Phased regression results of digital finance in different dimensions.

	df≤1.8110			1.8110 <df≤2.1843			df> 2.1843		
	(1)	(2)	(3)	(1)	(2)	(3)	(1)	(2)	(3)
	pol	pol	pol	pol	pol	pol	pol	pol	pol
df1	-0.330			-4.517***			-4.505***		
	(1.310)			(1.294)			(0.932)		
df2		-0.977			-5.389***			-7.020***	
		(1.365)			(0.780)			(1.216)	
df3			-0.028			0.627			-1.559*
			(0.732)			(1.346)			(0.801)

Standard errors are in parenthesis.

*** p<0.01,

** p<0.05,

* p<0.1.

**Fig 3:**
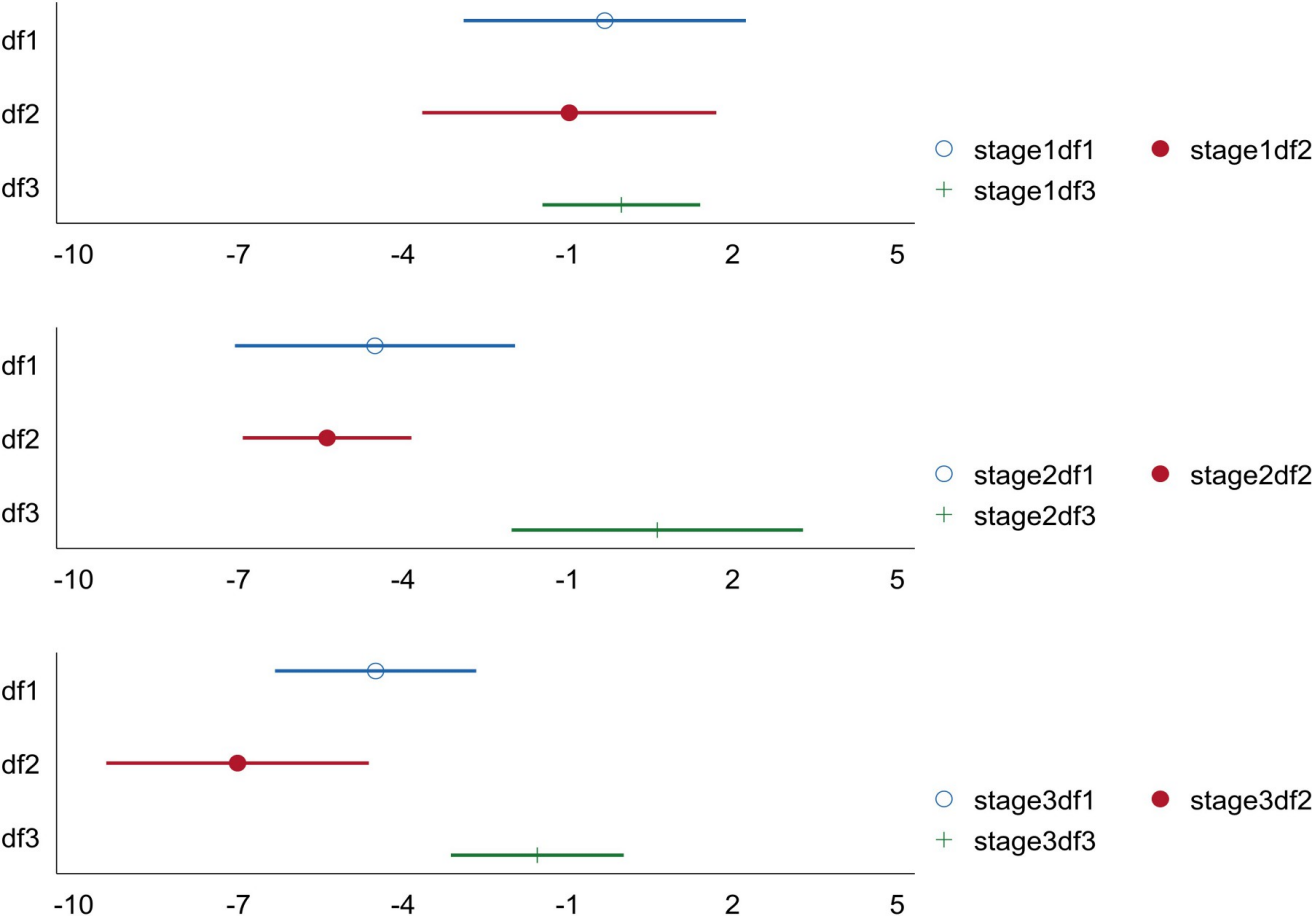
Marginal effects of three stages df1, df2 and df3 on pol in three stages.

As can be seen from Table 10 and Fig 3, when the development of digital finance is at the primary stage, digital finance cannot significantly reduce industrial pollution emissions in all three dimensions. When the development of digital finance is at the intermediate stage, its breadth of coverage (df1) and depth of use (df2) can significantly reflect the inhibitory effect on industrial pollution emissions. The phenomenon of the first acceleration of emission reduction effect appears. When the development of digital finance is at an advanced stage, the coefficients of breadth of coverage (df1), depth of use (df2) and degree of digital support (df3) are significantly negative at the same time. The absolute value of the coefficient of breadth of coverage (df1) decreases, but the absolute value of the coefficient of depth of use (df2) increases significantly, indicating that when the development of digital finance is at an advanced stage, digital finance can reduce industrial pollution emissions in all three dimensions and the depth of use plays a leading role. At this stage, the level of suppression of industrial pollution emissions by digital finance is further increased and then the second acceleration of the emission reduction effect occurs.

### 4.6 Robustness analysis

Although this paper tries to control the influence brought by relevant variables, there are many factors affecting industrial pollution and it is difficult to avoid the problem of omitted variables with the control variables that have been set so far. Therefore, this paper adopts instrumental variables to solve the possible endogeneity problem. In this paper, drawing on the approach of Xie et al. (2018), the ratio of the number of Internet broadband access subscribers to the year-end population in prefecture-level cities from 2002 to 2010 is chosen to carve out the local Internet penetration rate (rate_inter) and used as an instrumental variable [41]. On the one hand, Internet penetration, as a prerequisite for digital finance applications, satisfies the correlation condition as an instrumental variable. On the other hand, the historical digital finance Internet penetration status has almost no effects on industrial pollution emissions today. So, this variable can simultaneously satisfy the exclusion requirement as an instrumental variable. Meanwhile, to ensure the reliability of the study, this paper adopts the method of replacing the explained variables for robustness analysis and PM2.5 concentration is chosen to replace the sum of industrial pollutant emissions per unit area.

The results after regression through panel data are shown in Table 11. Columns (1) (2) report the results of the one-stage and two-stage regressions of the instrumental variables method. The Anderson LM test shows that there is no problem of under-identification of instrumental variables and the CDR test shows that there is no problem of weak instrumental variables, indicating that the instrumental variables method is valid and there is no change in the significance and sign of the main variable. Columns (3) (4) (5) are regressions with industrial wastewater (wwater), industrial sulfur dioxide (so2) and industrial dust (dust) as explained variables for the instrumental variable method, respectively. Their regression results show differences in the magnitude of coefficient values and no difference in significance and sign compared with the regression results in Table 5 in the previous paper. Column (6) shows the regression results after replacing the explained variables and there is no change in the significance and sign of the main variable. The above regression results indicate that the study findings are robust and reliable.

**Table 11:** Regression and robustness test results for instrumental variables.

	(1)	(2)	(3)	(4)	(5)	(6)
	fitst	pol	pol	pol	pol	pm
VARIABLES	df	pol	wwater	so2	dust	
rate_inter	1.941***					
	(0.162)					
df		-12.449***	-0.644***	-8.428***	-3.372**	-9.925***
		(1.912)	(0.092)	(0.717)	(1.657)	(0.359)
lnpgdp	1.487***	12.717***	0.626***	8.809***	3.279	-2.215***
	(0.037)	(3.162)	(0.153)	(1.185)	(2.740)	(0.799)
lnpopu	1.144***	18.813***	0.228	12.396***	6.145	-0.917
	(0.261)	(6.453)	(0.312)	(2.419)	(5.591)	(4.594)
innovation	-0.003***	0.031	0.000	0.005	0.026	-0.005
	(0.001)	(0.024)	(0.001)	(0.009)	(0.021)	(0.024)
lnfc	-0.087***	-0.785**	-0.047***	-0.441***	-0.297	-0.715***
	(0.011)	(0.318)	(0.015)	(0.119)	(0.275)	(0.196)
edu	0.030***	0.106	0.029***	0.157***	-0.081	-0.003
	(0.005)	(0.126)	(0.006)	(0.047)	(0.109)	(0.080)
Observations	2,340	2,340	2,340	2,340	2,340	2340
R-squared		0.036	0.070	0.067	0.008	0.486
Number of code	260	260	260	260	260	260
Anderson LM		134.854***	134.854***	134.854***	134.854***	
CDW		143.787***	143.787***	143.787***	143.787***	

Standard errors are in parenthesis

*** p<0.01,

** p<0.05,

* p<0.1.

## 5. Discussion

The empirical results for 260 cities in China from 2011–2019 show that digital finance can effectively reduce industrial pollution emissions and industrial structure plays a mediating role in this process. The pollution reduction effect of digital finance will gradually be strengthened with the continuous development of digital finance. The reasons, policies and recommendations are discussed next.

### 5.1 Impact of digital finance on industrial pollution emissions

The ability of digital finance to reduce industrial pollution emissions relies heavily on providing a favorable financing environment for the implementation of cleaner production in industrial enterprises that lack financial resources.

The realization of industrial pollution emission reduction requires the adoption of clean production methods and the implementation of circular resource utilization, which puts forward high requirements for financial support. For example, in 2020, Hefei Circular Economy Demonstration Park signed 25 projects, with a total investment of 32.16 billion yuan, including six projects of more than 1 billion yuan and five projects of 0.5–1 billion; 2011–2022, the Qinghai Provincial Development and Reform Commission has issued three batches of provincial circular economy development special funds investment plan, with a total of 2.416 billion yuan. However, due to the externalities and publicity of pollution abatement, only large industrial enterprises are likely to meet their funding needs through traditional financial services. For example, China’s heavy chemical industry is characterized by high consumption and high emissions, requiring the purchase of large amounts of expensive environmental protection production equipment. But the organization of chemical industry is fragmented and a large number of small and medium-sized enterprises, as the main players in the industry, cannot get financial support for purchasing environmental protection production equipment. In addition, there has been a trend in recent years for Chinese industrial enterprises to move to the mid-west, northeast and urban-rural areas, yet these areas tend to have relatively few financial resources, further reflecting the inadequate functioning of traditional finance in the process of reducing industrial pollution.

The application of digital finance provides a balanced investment and financing environment for industrial enterprises and establishes more channels, levels and forms of financing mechanisms. Compared with traditional finance, digital finance has more data and information due to its deep integration with technology and its way to meet financing needs is more flexible and personalized. Traditional finance is based on the credit, financial and operational status of enterprises and mostly serves large enterprises while digital finance can benefit a large number of small and medium-sized enterprises based on transaction records. Traditional finance uses the real estate of enterprises as collateral, while digital finance has the transaction data and logistics information of enterprises. The data can be used to personalize to assess risks and provide “data pledge”, which can offer support for clean production, energy conservation and emission reduction of industrial enterprises. For example, a bank in Zhejiang Province of China has used blockchain technology to build a "platform of receivables chain" to provide financial services to real enterprises through the Internet, transforming receivables into electronic tools of payment, settlement and financing, building more than 1,000 platforms for core enterprises, involving more than 3,000 upstream and downstream small and medium-sized enterprises, issuing receivables 78 billion yuan. Based on the "platform of receivables chain”, the bank and a petrochemical trading center and a forwarding company in the province jointly created a blockchain with warehouse receipt for oil products trading, which has the functions of warehouse receipt issuance, convenient warehouse receipt transfer, characteristic pledge financing and efficient bill of lading pickup, greatly easing the financial pressure of upstream and downstream small and medium-sized enterprises. Taking the enterprise financial service platform of a digital technology group in China as an example, the platform can provide financial services for enterprises in five ways: credit loan, prepayment financing, receivable financing, movable property financing and bill financing, among which the maximum amount of warehouse receipt pledge financing reaches 100 million RMB and the annual interest rate of credit loan can even be 3.6%.

Moreover, digital finance has improved the security and authenticity of information that provided more accurate and efficient services for financial supervision in terms of standard promotion, statistics, auditing and anti-greenwashing. Taking a bank in Zhejiang Province as an example, with the help of digital finance, from the end of 2019 to 2020, smart green identification and full process labeling have been carried out for about 4,000 credit businesses. The projects with environmental benefit can be recognized and supported preferentially. In addition, the data reporting was more effective because at least two-thirds of the cycle was compressed. By 2020, the system was further improved and upgraded in terms of green identification efficiency, environmental benefit measurement and ESG evaluation capability. Also, the system was replicated in other small and medium-sized banks in China.

### 5.2 Mediating effects of industrial structure

Industrial structure plays an intermediary role in the process of industrial pollution reduction by digital finance. The reason is that digital finance promotes the development of the tertiary industry, optimizes the industrial structure and reduces the industrial pollution brought by the secondary industry. In China, the national economy is still dominated by the secondary industry and the development of the tertiary industry lacks momentum. However, the larger output value of the secondary industry means more industrial pollution emissions. Digital finance plays a positive role in the optimization of industrial structure by alleviating the uneven allocation of financial resources, narrowing the income gap, promoting economic growth, innovation and entrepreneurship.

For example, digital finance can, on the one hand, improve the social credit environment and drive consumption upgrade so as to increase the demand of service industry; on the other hand, digital finance can improve the financial infrastructure, give rise to new business models so as to increase the supply of service industry, thus promoting the development of tertiary industry which mainly consists of service industry. Since Alipay, WeChat, UnionPay and other payment companies have cooperated deeply with e-commerce, consumer demand has been significantly enhanced, which has also given rise to a convenient payment industry. With the increase of consumers’ demand for convenience in bill payment, the scale of the bill payment industry has been steadily developed.

In addition, digital finance can play a crucial role in supporting the development of green industries through financial guidance. For example, in 2017, Chinese company Ant Financial and the United Nations Environment Programme officially launched the Green Digital Finance Alliance at the World Economic Forum in Davos, which attracts global financial technology partners to join. The alliance will further enable digital finance to adjust the industrial structure from the demand and supply sides, guide green consumption and green financing and promote the development of green industries. Another example is the Huzhou Green Finance Integrated Service Platform, which uses the Internet and big data technology to realize the information connection of nearly 10 departments. The platform consists of three major parts: "green identification of enterprises and projects", "enterprise and bank connection platform" and "enterprise and capital connection platform". The functions of this platform can help reduce the financing cost of green industries and improve the efficiency of financing so as to enhance the development level of local green industries.

### 5.3 The threshold effects of digital finance

There are two bottlenecks in the process of reducing industrial pollution by digital finance. When the bottleneck is broken, its industrial pollution reduction effect appears to be accelerated. The reason for this phenomenon may be related to the different characteristics of the stage of digital finance development process.

The development of digital finance in China has gone through three stages: initially, digital finance appeared in the form of Internet-enabled traditional financial institutions, followed by the significant growth of digital finance users because of fintech companies taking advantage of their own massive user base. Finally, the depth of use of digital finance has been significantly enhanced by traditional finance through in-depth cooperation with fintech companies. In the early stage of digital finance development, the breadth of coverage, depth of use and the degree of digital support were low, the application of digital finance by industrial enterprises was not common and the industrial emission reduction effect was not obviously reflected. When the development of digital finance breaks through the first bottleneck into the intermediate stage, the breadth of coverage and depth of use can significantly suppress industrial pollution emissions. The service model of digital finance fits with the needs of industrial enterprises to purchase environmental protection production equipment and enables more enterprises to achieve cleaner production. The level of industrial pollution emission reduction is significantly improved and the first acceleration of emission reduction effect occurs. When the development of digital finance breaks through the second bottleneck into the advanced stage, the financial services provided to industrial enterprises become more diversified. After the scale of financing expands, the industrial emission reduction function of the depth of digital finance which plays a leading role is more adequately reflected, thus further improving the level of suppression of industrial pollution emissions by digital finance so that the second acceleration of the emission reduction effect occurs.

For example, in China’s green finance comprehensive service platform, through big data and cloud computing technology to empower financial institutions, the capacity of green financial services was enhanced, the scale of services provided by digital finance to industrial enterprises was expanded. The initial effect of digital finance on industrial pollution emission reduction was manifested. After that, the depth of digital finance use was strengthened and the circular economy public service platform applied digital finance to the development of circular economy in industrial parks and enterprises. In addition to building a financing platform, the provincial-level industrial symbiosis and waste exchange and trading system were created. The level of suppression of industrial pollution emission by digital finance was further improved.

### 5.4 Policy and future direction

In China’s 12th and 13th Five-Year Plans, it is mentioned that "eliminating backward production capacity, making full use of technological innovation, saving resources and energy and upgrading clean production". After the release of the 14th Five-Year Plan, "energy saving" and "clean production" are still important ways to develop green industry and reduce industrial pollution. At the same time, the application of financial technology in green finance will continue to strengthen the inhibiting effect of digital finance on industrial pollution emissions.

In the future, the application of digital finance in industrial enterprises will become more common in order to provide financial support for the realization of clean production and circular economy. Furthermore, the industrial pollution reduction function of digital finance will be amplified with the further development. With the adjustment to the industrial structure by digital finance, innovative business models will be created so as to promote industrial structure optimization.

## 6. Conclusions and recommendations

Through the empirical analysis of panel fixed effects model, mediating effects model and threshold effects model, this paper draws the following conclusions: (1) Digital finance can significantly reduce industrial pollution emissions and has a significantly inhibitory effect on industrial wastewater, industrial sulfur dioxide and industrial dust emissions. Strengthening the depth of use of digital finance can better play the industrial pollution reduction function of digital finance. (2) Industrial structure plays an intermediary role in the process of reducing industrial pollution by digital finance, which reduces industrial pollution by optimizing industrial structure and promoting the development of tertiary and green industries. (3) The impact of digital finance on industrial pollution emission has double threshold effects. When the development of digital finance breaks through the threshold value, the industrial pollution emission reduction effect appears to accelerate.

Based on the above empirical results and theoretical analysis, this paper puts forward the following policy recommendations: (1) Digital finance can break through the limitations of geographic space and economic level, regulate the use of funds, strengthen the effectiveness of policies and reduce industrial pollution. The government should improve the depth of the use of digital finance, especially in areas with serious industrial pollution and lack of financial resources. Meanwhile, making full use of digital finance can revitalize industrial enterprises’ assets and provide financial support for industrial enterprises to realize clean production and circular economy. (2) The role of digital finance in supporting the optimization of industrial structure and green finance should be strengthened. By giving birth to new service industries and green-oriented consumption, the development of the tertiary industry can be promoted and the industrial pollution can be reduced through digital finance. (4) Digital finance improves the security and authenticity of information so it should be applied extensively in the promotion of standards, statistics, auditing and anti-greenwashing so that the accuracy of the effect of digital finance on industrial pollution can be improved.

## Supporting information

S1 Appendix Digital financial inclusion indicator system. (DOCX)

S2 Appendix Phased regression results of digital finance in different dimensions. (DOCX)
