# Supplementary material for: Can digital finance reduce industrial pollution? New evidence from 260 cities in China
Source: PLoS One. 2022 Apr 14;17(4):e0266564. doi: 10.1371/journal.pone.0266564 (PMC9009666; doi:10.1371/journal.pone.0266564)
Supplement: S1 Appendix — (DOCX) [file pone.0266564.s001.docx]

**Appendix Table. 1 Digital Financial Inclusion Indicator System.**

| Level 1 Dimension | Secondary Dimension | Specific indicators |
| --- | --- | --- |
| Breadth of coverage | Account Coverage | Number of Alipay accounts per 10,000 people |
|  |  | Proportion of Alipay tied card users |
|  |  | Average number of bank cards tied to each Alipay account |
| Depth of use | Payment Business | Number of payments per capita |
|  |  | Amount paid per capita |
|  |  | High frequency (active 50 times a year and above) active users as a percentage of active 1 time a year and above |
|  | Money Fund Business | Number of balance purchases per capita |
|  |  | Per capita purchase balance amount |
|  |  | Number of Alipay users per 10,000 purchasing balance |
|  | Lending business  (To individual business) | Number of Internet consumer loans per 10,000 adult Alipay users |
|  |  | Number of loans per capita |
|  |  | Loan amount per capita |
|  | Credit business  (for micro and small operators) | Number of Internet micro and small business loans per 10,000 adult Alipay users |
|  |  | Average number of loans per household for micro and small operators |
|  |  | Average loan amount for small and micro operators |
|  | Insurance Business | Number of insured users per 10,000 Alipay users |
|  |  | Number of insurance strokes per capita |
|  |  | Amount of insurance per capita |
|  | Investment Business | Number of Alipay users per 10,000 people involved in Internet investment and wealth management |
|  |  | Number of investments per capita |
|  |  | Investment amount per capita |
|  | Credit Business | Number of people using credit-based lifestyle services per 10,000 Alipay users |
|  |  | Number of calls per capita for natural person credit |
| Degree of digital support services | Mobility | Percentage of mobile payment transactions |
|  |  | Percentage of mobile payment amount |
|  | Affordability | Average loan interest rate for small and micro operators |
|  |  | Average personal loan interest rate |
|  | Crediting | Percentage of payments made by chanting |
|  |  | Chanting payment amount accounted for |
|  |  | Percentage of Sesame Credit free deposit (compared to all cases requiring a deposit) |
|  |  | Sesame Credit free deposit amount accounted for (more than all need deposit situation) |
|  | Facilitation | Percentage of user QR code payments |
|  |  | Percentage of the amount of user QR code payment |
