# Supplementary material for: Can digital finance reduce industrial pollution? New evidence from 260 cities in China
Source: PLoS One. 2022 Apr 14;17(4):e0266564. doi: 10.1371/journal.pone.0266564 (PMC9009666; doi:10.1371/journal.pone.0266564)
Supplement: S2 Appendix — (DOCX) [file pone.0266564.s002.docx]

**Appendix Table. 2 Phased regression results of digital finance in different dimensions.**

|  | (1) | (2) | (3) | (4) | (5) | (6) | (7) | (8) | (9) |  |
| --- | --- | --- | --- | --- | --- | --- | --- | --- | --- | --- |
|  | pol | pol | pol | pol | pol | pol | pol | pol | pol |  |
| df1 | -0.330 |  |  | -4.517*** |  |  | -4.505*** |  |  |  |
|  | (1.310) |  |  | (1.294) |  |  | (0.932) |  |  |  |
| df2 |  | -0.977 |  |  | -5.389*** |  |  | -7.020*** |  |  |
|  |  | (1.365) |  |  | (0.780) |  |  | (1.216) |  |  |
| df3 |  |  | -0.028 |  |  | 0.627 |  |  | -1.559* |  |
|  |  |  | (0.732) |  |  | (1.346) |  |  | (0.801) |  |
| lnpgdp | -0.016 | 0.943 | -0.640 | -0.485 | 0.646 | -0.717 | -1.577 | 1.225 | -1.492 |  |
|  | (3.738) | (3.373) | (3.397) | (0.524) | (0.520) | (0.535) | (1.157) | (1.231) | (1.187) |  |
| lnpopu | 7.302 | 7.404 | 6.996 | -0.127 | -1.116 | 0.419 | 4.150 | 8.774 | -4.931 |  |
|  | (15.338) | (15.292) | (15.292) | (7.092) | (6.568) | (7.300) | (7.030) | (7.092) | (7.236) |  |
| innovation | 0.047 | 0.045 | 0.048 | 0.019 | 0.035 | 0.033 | 0.019 | 0.021 | 0.021 |  |
|  | (0.056) | (0.056) | (0.056) | (0.030) | (0.027) | (0.030) | (0.014) | (0.014) | (0.014) |  |
| lnfc | 0.023 | 0.020 | 0.021 | 0.076 | 0.180 | 0.199 | 0.054 | 0.074 | 0.100 |  |
|  | (0.578) | (0.578) | (0.578) | (0.183) | (0.167) | (0.186) | (0.269) | (0.266) | (0.276) |  |
| edu | -0.140 | -0.137 | -0.146 | 0.768*** | 0.745*** | 0.760*** | 0.020 | -0.008 | 0.013 |  |
|  | (0.227) | (0.226) | (0.226) | (0.186) | (0.172) | (0.192) | (0.076) | (0.075) | (0.078) |  |
| _cons | -31.781 | -41.623 | -23.729 | 11.154 | 5.591 | -1.174 | 5.418 | -46.433 | 52.257 |  |
|  | (93.158) | (89.877) | (89.902) | (40.300) | (37.208) | (41.429) | (40.652) | (43.211) | (41.849) |  |
| Obs. | 1257 | 1257 | 1257 | 467 | 467 | 467 | 616 | 616 | 616 |  |
| R-squared | 0.002 | 0.002 | 0.002 | 0.147 | 0.268 | 0.096 | 0.079 | 0.102 | 0.031 |  |
|  | | | | | | | | | | |
| Standard errors are in parenthesis | | | | | | | | | | |
| *** p<0.01, ** p<0.05, * p<0.1 | | | | | | | | | | |
